# SHP-1 Arrests Mouse Early Embryo Development through Downregulation of Nanog by Dephosphorylation of STAT3

**DOI:** 10.1371/journal.pone.0086330

**Published:** 2014-01-21

**Authors:** Songna Yin, Haibo Wu, Jiaxing Lv, Xinying Wu, Yan Zhang, Juan Du, Yong Zhang

**Affiliations:** 1 College of Veterinary Medicine, Northwest A&F University, Yangling, Shaanxi, China; 2 Key Laboratory of Animal Biotechnology, Ministry of Agriculture, Northwest A&F University, Yangling, Shaanxi, China; 3 College of Life Sciences, Northwest A&F University, Yangling, Shaanxi, China; Cardiological Center Monzino, Italy

## Abstract

Src-homology protein tyrosine phosphatase-1 (SHP-1) is a protein tyrosine phosphatase that is implicated in the regulation of growth, differentiation, survival, apoptosis and proliferation of hematopoietic cells and other cell types. Here, we found that SHP-1 is involved in regulation of early embryonic development. Embryos overexpressing SHP-1 were mainly arrested at the 8-cell stage, and Nanog mRNA expression was first observed in the morulae that showed down-regulation of SHP-1. These results suggested an antagonistic relationship between SHP-1 and Nanog during early embryonic development. Next, the specific mechanism was examined in mouse F9 embryonal carcinoma cells. We confirmed that signal transducer and activator of transcription 3 (STAT3) was a substrate for SHP-1 by co-immunoprecipitation. Using overexpression and knockdown strategies, we found that SHP-1 participated in regulation of Nanog expression. Furthermore, site mutation of STAT3 was performed to confirm that SHP-1 was responsible for rapid STAT3 dephosphorylation and a decrease of Nanog expression in F9 cells. These findings suggest that SHP-1 plays a crucial role during early embryonic development. Thus, SHP-1 may function as a key regulator for Nanog that specifically demarcates the nascent epiblast, coincident with the domain of X chromosome reprogramming.

## Introduction

Src-homology protein tyrosine phosphatase-1 (SHP-1) is a cytokine-inducible SH2-containing protein. It has been implicated in the regulation of multiple signaling pathways involved in cell growth, differentiation, survival and apoptosis [1], [2]. SHP-1 is a non-receptor phosphatase that negatively regulates cytokine signaling and tyrosine kinase receptors, and functionally interacts with numerous proteins through SH2-mediated association with the phosphorylated tyrosine subunit [3]. Moreover, SHP-1 inhibits activated STAT3 in six human T-cell leukemia virus-1 (HTLV-1)-negative and seven HTLV-1-positive leukemic T-cell lines [4]–[6]. Nanog is a pivotal homeodomain transcription factor that maintains the pluripotency of embryonic stem (ES) cells and blocks their differentiation into endodermal cells. In the embryo, Nanog is essential for early embryonic development [7]. Nanog mRNA expression is first observed in the morulae, which is down-regulated prior to implantation. It is also restricted to the epiblast and excluded from the primitive endoderm. Down-regulation of Nanog during the implantation stage is consistent with the intimate association with restricted expression to the epiblast [8]. Nanog is critical for the early inner cell mass to mature into the pluripotent epiblast [9]. Furthermore, Nanog specifically demarcates the nascent epiblast, coincides with the domain of the X chromosome. Without Nanog expression, pluripotency does not develop and the inner cell mass is arrested in a pre-pluripotent indeterminate state that is ultimately nonviable [9]. A previous study showed that phosphorylated STAT3 binds to the murine Nanog promoter and activates its transcription [10]–[13]. STAT3 is activated by phosphorylation at tyrosine residues, leading to its dimerization via reciprocal interactions between the SH2 domain of one monomer and the phosphorylated tyrosine of the other, causing further activation of target gene transcription [14]. In this study, we found that SHP-1 has an effect on blastocyst formation. We also describe the mechanism of SHP-1 signaling through STAT3 following stimulation, which decreases Nanog expression by blocking STAT3 activation in F9 cells.

## Materials and Methods

### Ethics Statement

All procedures and experiments were approved by the Animal Care Commission of the College of Veterinary Medicine, Northwest A&F University and are in accordance with the National Institutes of Health Guide for the Care and Use of Laboratory Animals. Adult male and female ICR strain mice were purchased from the Experimental Animal Center of The Xi’an Jiaotong University (Xi’an, China). They were maintained on a 14/10 h light/dark cycle with free access to food and water in the Laboratory Animal Facility of the College of Veterinary Medicine, Northwest A&F University.

### Antibodies and Reagents

Unless otherwise indicated, reagents were purchased from Sigma Chemical Co. (St. Louis, MO, USA). Rabbit anti-SHP-1 was obtained from Santa Cruz Biotechnology (Santa Cruz, CA, USA), and a mouse anti-Nanog polyclonal antibody was obtained from Bethyl Laboratories (Montgomery, TX, USA). Rabbit anti-STAT3 antibody was purchased from Merck Millipore (Millipore Chemicals, MA, USA) and a rabbit anti-phospho-STAT3 antibody was purchased from Cell Signaling Technology (Beverly, MA, USA). Mouse anti-GAPDH polyclonal antibody was purchased from beyotime (Beyotime, Shanghai, China). Secondary antibodies for ICC were alexa fluor 488-labeled goat anti-Rabbit IgG and cy3-labeled goat anti-mouse IgG purchased from beyotime (Beyotime, Shanghai, China). Secondary antibodies for Western were goat anti-rabbit IgG-HRP and goat anti-mouse IgG-HRP purchased from beyotime (Beyotime, Shanghai, China).

### Cell Culture and Transient Transfection

Mouse F9 cells were obtained from ATCC (Manassas, V A, USA) and were cultured on 0.2% gelatin-coated dishes in Dulbecco’s modified Eagle’s medium (DMEM; Invitrogen, USA) supplemented with 10% fetal calf serum (Gibco, Invitrogen) at 37°C/5% CO_2_. Transfections were performed using Lipofectamine 2000 reagent (Invitrogen) following the manufacturer’s recommended protocols. F9 cells were seeded into 6-well plates at least 24 h before transfection at a density of 2×10^5^ cells/well. Cells were then transfected with a plasmid-Lipofectamine 2000 mix, and 4–6 h post-transfection the medium was changed. Cells were transfected with pCMV-MYC as a negative control.

### Vector Construction

The Nanog promoter was cloned from F9 genomic DNA and ligated into the pGL4.10 luciferase reporter vector, and confirmed by DNA sequencing. The Np-WT Nanog promoter reporter plasmid contains the Nanog proximal promoter region from −770 bp – +294 bp (relative to transcription start site) and the region from −5066 bp – −4853 bp which contains the STAT3 binding sequence (TTCCTAGAA), the core STAT3 binding sequence (TTCCTAGAA) mutated to CCACTAAGG was set as Np-MUT. Full-length SHP-1 was amplified from F9 cDNA and ligated into pCMV-MYC. Two different transcript variants of SHP-1 were constructed and used in this study (pCMV-MYC-SHP-1-V1/V2), and both were confirmed by DNA sequencing. The full-length STAT3 was amplified from F9 cDNA and ligated into pCMV-MYC to overexpress STAT3. Mutated STAT3, phospho-mimetic mutant (Y705D), or dominant negative forms of STAT3 (Y705F) were ligated into pCMV-MYC to produce two separate plasmids. The primers used for plasmid construction are listed in Table S1.

### Reverse Transcription Polymerase Chain Reaction (RT-PCR) and Quantitative Real-time PCR (qPCR)

Total RNA was isolated from F9 cells using TRIzol reagent (Invitrogen) following the manufacturer’s instructions. First strand cDNA was generated from total RNA using a SYBR PrimeScript RT-PCR Kit (Takara, Japan). Embryos were lysed and first-strand cDNA was synthesized directly using SuperScript III CellsDirect cDNA synthesis kits (Invitrogen, CA, USA) according to the manufacturer’s instructions. The qPCR assays were carried out using an ABI StepOnePlus PCR system (Applied Biosystems, CA, USA) under the following conditions: 95°C for 30 s; followed by 40 cycles of 95°C for 5 s, and 60°C for 30 s. We used SYBR Premix ExTaq II (Takara), and the Ct method was used to calculate the relative quantity of the target mRNA. Results were normalized to beta-actin, relative to the calibrator, and expressed as fold-change (2^–ΔΔCt^) [15]. All the experiments were repeated three times; the primers we used for qPCR are listed in Table S2.

### Short Interfering (si) RNA-mediated Knockdown

Commercial SHP-1 siRNA (m):sc-29479, commercial Nanog siRNA (m) sc-44833, commercial STAT3 siRNA (m) sc-29494 were purchased from Santa Cruz Biotechnology (Santa Cruz, CA, USA) and resuspended in RNAase-free water (Santa Cruz Biotechnology) following the manufacturer’s instructions. Transfection of F9 cells were conducted with Lipofectamine RNAiMAX transfection reagent (Invitrogen) according to the manufacturer’s instructions. F9 cells were seeded into 6-well plates at least 24 h before transfection at a density of 2×10^5^ cells/well. The siRNA (10 µM)/Lipofectamine RNAiMAX mix was added to cells, 4–6 h after transfection, and the medium changed. Cells treated with a non-specific siRNA served as a negative control.

### Luciferase Assays

Luciferase measurements were conducted with the Dual Luciferase Reporter (DLR) Assay System (Promega, WI, USA) according to the manufacturer’s instructions. F9 cells were transfected with the aforementioned reporter constructs, and a Renilla luciferase plasmid (pRL-SV40) was co-transfected as an internal control. At 24 h post-transfection, cells were lysed with 200 µL of passive lysis buffer for 15 min with shaking. We then transferred 20 µL of each lysate to a 96-well plate and assayed through the addition of 100 µL of Luciferase Assay Reagent and 100 µL of Stop & Glo Reagent. Data were collected with a VICTOR X5 Multilabel Plate Reader (PerkinElmer, USA). The relative activities of the promoters were measured by firefly luciferase luminescence divided by Renilla luciferase luminescence. Sequences of primers for Nanog promoters are listed in Table S3.

### Western Blotting

Cells were lysed with RIPA buffer [50 mM Tris pH 7.8, DTT 1 mM, 10 mM MgCl_2_, 70 mM KCl, 0.5% NP-40, 0.1% (w/v) SDS] and supplemented with protein inhibitor cocktail tablets (Roche Diagnostics). Lysates were centrifuged (12,000×*g*, 10 min, 4°C), and the supernatants assayed for protein concentration using a BCA Protein Assay kit (Pierce, Rockford, IL, USA). Supernatants were subjected by sodium dodecyl sulfate polyacrylamide gel electrophoresis (SDS-PAGE) and then transferred to PVDF (Millipore) membranes. Membranes were blocked with Tris-buffered saline containing Tween-20 and 5% (w/v) fat-free milk powder, and then incubated with primary antibodies at 4°C overnight followed by horseradish peroxidase-conjugated secondary antibodies.

### Co-immunopreciptation

Lysates were harvested from F9 cells using ice-cold non-denaturing lysis buffer (Thermo Scientific, Rockford, IL, USA). Cell lysates were incubated with rabbit anti-SHP-1 or rabbit anti-STAT3 antibodies overnight, with gentle shaking. Pierce protein A/G beads were added and incubated at 4°C for 3 h. The beads were washed twice with 1×IP buffer and resuspended in 1×SDS loading buffer. A rabbit IgG antibody was used as a negative control.

### Chromatin Immunoprecipitation (ChIP)

The F9 cells were cross-linked with 1% (w/v) formaldehyde for 10 min at room temperature and the reaction subsequently quenched with 125 mM glycine. Cell lysates were prepared, Genomic DNA was isolated and sheared to average lengths of 300–500 bp by ultrasonic and immunoprecipitated with anti-STAT3(Millipore Chemicals, MA, USA) antibodies. A 200-bp DNA fragment containing STAT3-binding sites was amplified by PCR. Fold-enrichment was determined by normalizing threshold cycle values of STAT3 ChIP against IgG ChIP. Primer sequences used for ChIP-qPCR are listed in Table S4 [16].

### Collection and Culture of Embryos

Zygotes were obtained from 8–10-week-old female mice superovulated with 10 IU pregnant mare serum gonadotropin and 10 IU human chorionic gonadotropin. After 48 h, these mice and mated with males. Zygotes were released from the ampullae of oviducts at 20 h after human chorionic gonadotropin administration and cumulus cells were removed by treatment with 1 mg/ml hyaluronidase and pipetting in H-KSOM. 2-, 4- and 8-cell morulae and blastocysts were collected by flushing oviducts with H-KSOM. Embryos were cultured in groups of 30–50 in microdrops (100 µl) of KSOMaa medium [17] supplemented with 4 mg/ml bovine serum albumin (BSA) under liquid paraffin oil in a humidified atmosphere with 5% CO_2_ at 37°C [18], [19].

### Electroporation of Plasmids or siRNA into Embryos

For electroporation, pCMV-MYC-SHP-1-V1/V2 and pCMV-MYC was purified by routine methods and diluted in Opti-MEM (Invitrogen, Carlsbad, CA, USA) at a concentration of 40 mg/ml. Then, a suitably sized drop (30 µl) of the diluted plasmid DNA was added between the electrodes fixed in an electroporation chamber. Zona-weakened embryos were linearly arranged in the flat chamber [19]. The siPORT Amine Transfection Agent (Ambion, TX, USA) was diluted in Opti-MEM (Invitrogen) medium and incubated for 10 min at RT. The scramble or the Nanog siRNA was diluted into Opti-MEM (Invitrogen) medium, then combined with the diluted siPORT Amine Transfection Agent and incubated for 10 min at RT. The zona-weakened embryos were mixed with the transfection complexes and linearly arranged in the flat chamber [20], simultaneously, the cell fusion instrument (ECM 2001; BTX Inc. CA, USA) was switched on. Electroporation conditions were DC 20 volts/1 ms pulse length/3 pulses/0 repeats [21]. After electroporation, embryos were washed three times and cultured in KSOMaa-BSA for 1.5 days [20].

### Immunohistochemistry

Murine pre-implantation embryos were fixed with 4% buffered paraformaldehyde solution for 15 min at RT. Embryos were exposed to 0.25% Triton X-100 and then incubated with primary antibodies overnight at 4°C. Following a wash with phosphate buffered saline, cells were incubated with secondary antibodies and then observed and photographed with the aid of a Nikon confocal microscope.

### Statistical Analysis

The results are presented as the mean±SD. Data were analyzed by one-way ANOVA and LSD tests using the SPSS13.0 software (SPSS Inc, Chicago, IL, USA).The difference was considered statistically significant at P<0.05.

## Results

### SHP-1 arrests Early Embryonic Development

**Figure 1:**
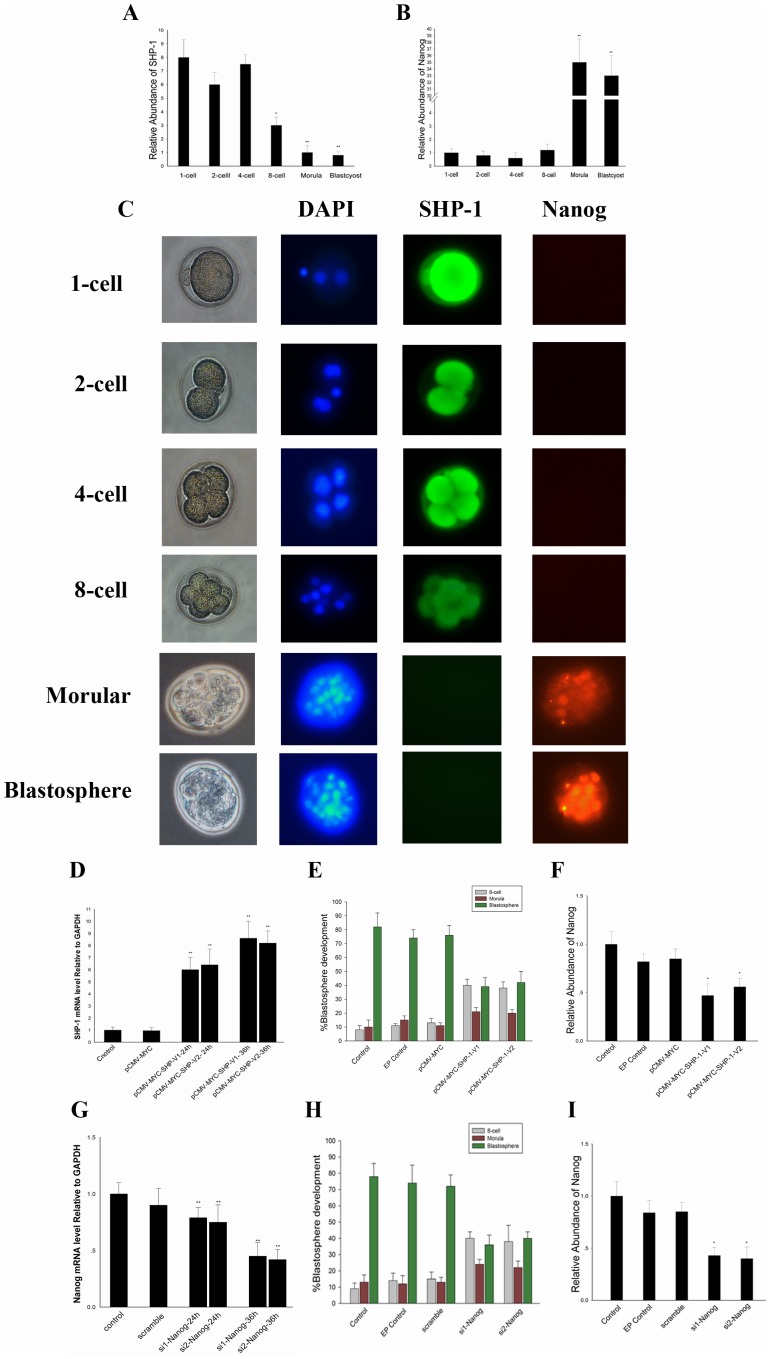
SHP-1 arrest early embryonic development via downregulated Nanog. A–B: The mRNA of SHP-1(A) and Nanog (B) at precisely defined stages from the Zygote until the late blastocyst stage. C: Immunofluorescence was performed to detect SHP-1 and Nanog in mouse pre-implantation, each sample was counterstained with DAPI (blue) to visualise DNA, anti-NANOG (red) and anti-SHP-1 (green) antibodies(Original magnification ×200). D:The transfection efficacy in this experiment was tested by observing the SHP-1 expression after overexpression. E: The SHP-1 overexpressed embryos were mainly arrested at the 8-cell stages. F: The mRNA of Nanog in SHP-1 overexpressed embryos. G: The Nanog expression after silencing. H: The Nanog knockdown embryos were mainly arrested at the 8-cell stages. I: The mRNA of Nanog in Nanog knockdown embryos. All data are presented as the mean +/− SD and are derived from three independent experiments. *P<0.05; **P<0.01; EP control: electroporated control.

SHP-1 has been implicated in the regulation of a multiplicity of cell signaling pathways [2], but the function of SHP-1 in embryos is relatively unknown. In the pre-implantation embryo, Nanog expression was detected in the morula and blastocyst, which was down-regulated after implantation. Down-regulation of Nanog may be critical to restrict expansion of the epiblast [22]. To prove this, we analyzed the distribution of SHP-1 and Nanog mRNA at precisely defined stages from the zygote until the late blastocyst stage during pre-implantation (Fig. 1A, B). The immunohistochemistry results showed that Nanog was first expressed in the morulae where SHP-1 expression was down-regulated and its expression in the blastocyst was confined to the inner cell mass but absent from the trophectoderm (Fig. 1C). To elucidate the function of SHP-1 in early embryonic development, SHP-1 was over-expressed in 8-cell embryos; the transfection efficacy in this experiment was tested by observing the gene expression after overexpression or silencing (Fig. 1D, G). As a result, embryos electroporated with pCMV-MYC-SHP-1-V1/V2 appeared to be arrested, whereas embryos in the control group reached the blastocyst stage. Embryos over-expressing SHP-1 were mainly arrested at the 8-cell stage (Fig. 1E). The Nanog mRNA of each group was also detected (Fig. 1F). Similarity, as we knockdown Nanog in 8-cell, the embryos were mainly arrested at the 8-cell stage (Fig. 1H, I). The results above indicated that SHP-1 can affect early embryonic development, and Nanog may involve in this regulatory network.

### SHP-1 Regulates Nanog Expression in F9 Cells

**Figure 2:**
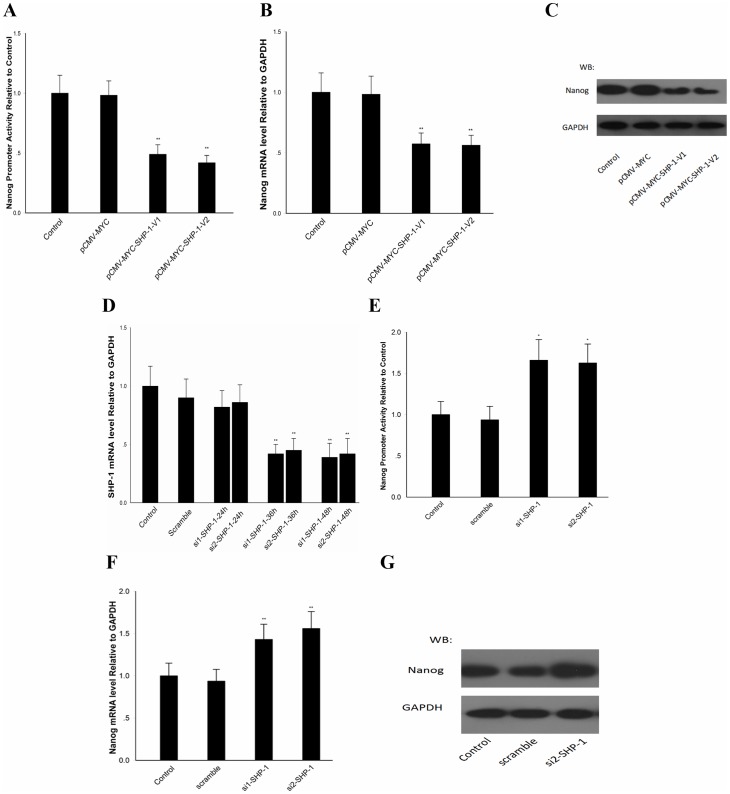
SHP-1 regulates Nanog expression in F9 cells. F9 cells transfected with pCMV-MYC-SHP-1-V1/V2, DLR assays, qPCR and western blotting were performed to detect the Nanog promoter activity (A) mRNA levels (B) and protein level (C); F9 cells transfected with si-SHP-1, the SHP-1 expression after silencing was detected (D) and DLR assays, qPCR and western blotting were performed to detect the Nanog promoter activity (E)mRNA levels (F) and protein levels (G). All data are presented as the mean +/− SD and are derived from three independent experiments. *P<0.05; **P<0.01; WB: western blot.

Due to the self-limitation of embryos, we decided to use F9 cell as a vitro model to investigate the specific mechanism of SHP-1 regulation of Nanog expression, since F9 cell has been widely used as a model for the analysis of the molecular mechanisms of differentiation and proliferation. DLR assays were conducted to detect Nanog promoter activity as previously described. In our results, Nanog promoter activity in F9 cells was reduced to about 40% when SHP-1 was overexpressed. Conversely, Nanog promoter activity was up-regulated around 1.55–1.62-fold when SHP-1 was knocked down (Fig. 2A, E). The SHP-1 expression after silencing is show in Fig. 2D. The RT-PCR and western blotting results showed that Nanog was also reduced at mRNA (55%) (Fig. 2B) and protein levels (Fig. 2C) when SHP-1 was overexpressed. Nanog was up-regulated at mRNA (1.5-fold) (Fig. 2F) and protein levels (Fig. 2G) when SHP-1 was knocked down. Taken together, these data suggested that SHP-1is participating in the regulation of Nanog in F9 cells.

### SHP-1 Prevents STAT3 Activation

**Figure 3:**
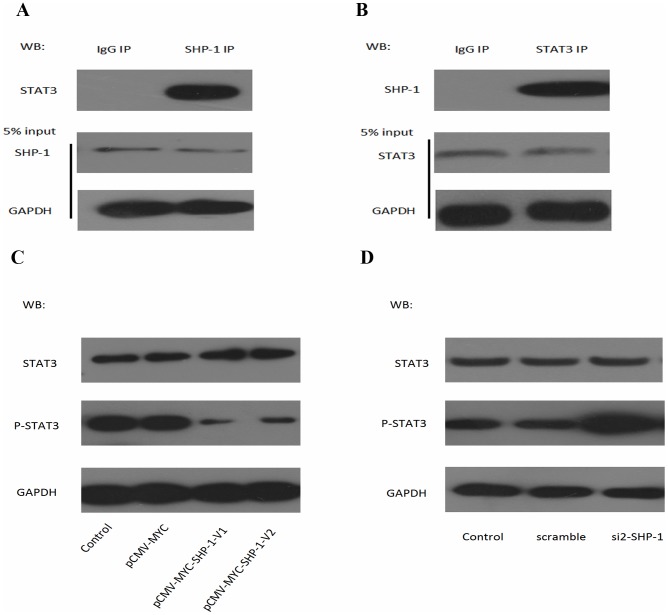
SHP-1 prevented STAT3 activation via dephosphorylates STAT3. A–B: The whole-cell lysates (input) and immunoprecipitated proteins were separated by 10% SDS-PAGE, followed by western blot with anti–SHP-1, anti–STAT3, or anti-GAPDH antibodies. Anti–SHP-1 immunoprecipitates was immunoprecipitated with polyclonal antibodies recognizing STAT3 (A) and immunoprecipitates was immunoprecipitated with polyclonal antibodies recognizing SHP-1 (B), a rabbit IgG antibody was used as a negative control. (C): Protein level of phosphorylation of STAT3 in F9 cells transfected with pCMV-MYC-SHP-1-V1/V2. (D): Protein level of phosphorylation of STAT3 in F9 cells transfected with SHP-1 siRNA. GAPDH was set as loading control. WB: western blot; p-STAT3: phospho-STAT3.

As previous studies have shown that SHP-1 is widely accepted as an inhibitor of STAT3 [23], we conducted the co-immunoprecipitation experiment to verified the interaction between SHP-1 and STAT3. Anti-STAT3 antibody was used to detect STAT3 in anti-SHP-1 immunoprecipitates [6], [24], [25]. An 89-kDa band corresponding in size to STAT3 was observed (Fig. 3A). Anti-SHP-1 antibody was used to detect SHP-1 in anti-STAT3 immunoprecipitates (Fig. 3B). These results show that STAT3 is a potential substrate for SHP-1. To have a better understanding on the biological importance of SHP1 in F9 cells, SHP-1-V1/V2 was overexpressed using pCMV-MYC- SHP-1-V1/V2. At 48 h post-transfection, western blotting analysis was conducted, phosphorylated STAT3 was significantly down-regulated when SHP-1-V1/V2 was overexpressed. Conversely, phosphorylated STAT3 was up-regulated when SHP-1 was inhibited by siRNA (Fig. 3C, D). Based on these data, we got a conclusion that SHP-1 can regulate STAT3 through dephosphorylation.

### STAT3 Binds to the Nanog Promoter and Activates Nanog Transcription

**Figure 4:**
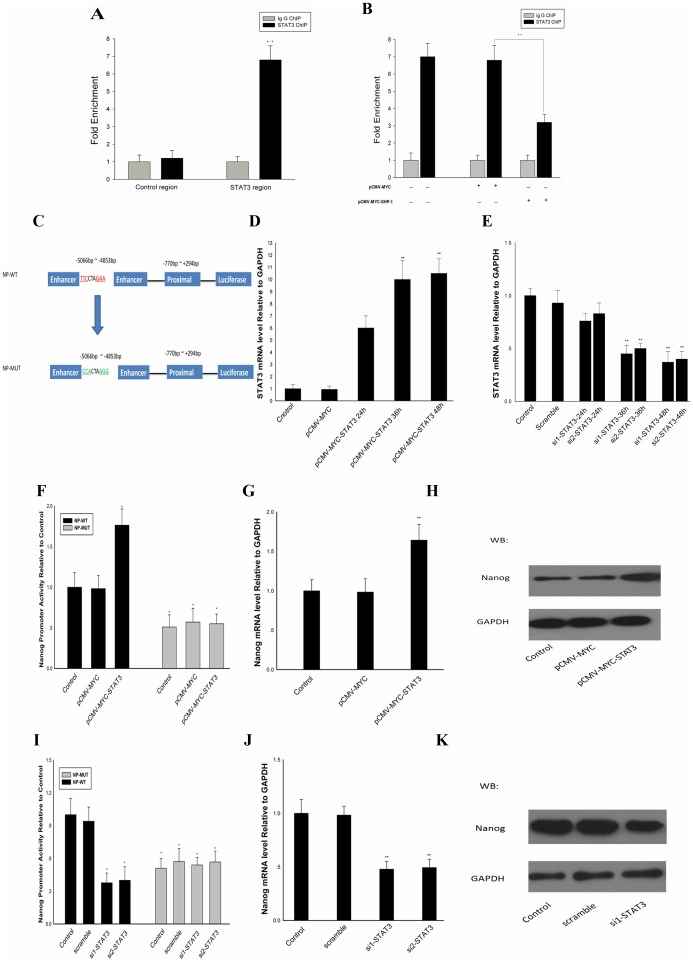
STAT3 binds to Nanog promoter and activates Nanog transcription. (A) ChIP assay was performed to confirm the binding of STAT3 to specific region of Nanog promoter. (B) ChIP in the presence of SHP-1 overexpression is also preformed. Overexpress SHP-1 can decrease the level of STAT3 binds to the Nanog promoter. (C): The Schematic representation of Np-WT and Np-MUT promoters reporter constructs. (D) STAT3 expression after overexpression. (E) STAT3 expression after silencing. F9 cells were transfected with Np-WT or Np-MUT and pCMV-MYC-STAT3 or STAT3 siRNA. F9 cells transfected with pCMV-MYC-STAT3, cell lysates were harvested for DLR assays (F), Values were normalized with normal cultured WT group, qPCR and western blotting were performed to detect the Nanog mRNA levels (G) and protein levels (H). F9 cells transfected with STAT3 siRNA,DLR assays, qPCR and western blotting were performed to detect the Nanog promoter activity (I) mRNA levels (J) and protein levels (K). All data are presented as the mean +/− SD and are derived from three independent experiments. *P<0.05; **P<0.01; GAPDH was set as loading control. WB: western blot; WT: wild type; MUT: mutation. ChIP: Chromatin immunoprecipitation.

It has been proved that in ES cells STAT3 can regulate the expression of Nanog by interaction with the promoter of Nanog [12], we conducted ChIP assays. As shown in Fig. 4A, a 6-fold enrichment of STAT3 was observed, Chip in the presence of SHP-1 overexpression is showed in Fig. 4.B, these data collectively indicate that, in F9 cells, STAT3 can binds to the Nanog promoter and activates the expression of Nanog, overexpressing SHP-1 can decrease the level of STAT3 binding to Nanog promoter. The Schematic representation of NP-WT and NP-MUT promoters is showed in Fig. 4. C. STAT3 expression after overexpression or silencing is showed in Fig. 4.D and Fig. 4E. In the NP-WT group, Nanog promoter activity was up-regulated 1.75-fold when STAT3 was overexpressed, in the NP-MUT group, overexpression of STAT3 had no effect on Nanog promoter activity (Fig. 4F).Western blotting and RT-PCR analysis indicated that there is a significant (1.6–1.7-fold) up-regulation of the mRNA levels of Nanog when STAT3 was overexpressed. Conversely, if endogenous STAT3 was degraded by si-STAT3, Nanog promoter activity was 35% compared with the control. In the NP-MUT group, knockdown of STAT3 had no effect on Nanog promoter activity (Fig. 4I). Western blotting and RT-PCR analysis indicated a 0.5–0.6-fold down-regulation of Nanog mRNAs when STAT3 activation was blocked (Fig. 4J, K). All these data indicate that the STAT3 binds to the Nanog promoter and activates the expression of Nanog in F9 cells.

### SHP-1 Regulates Nanog Expression via STAT3 Signal Pathway

**Figure 5:**
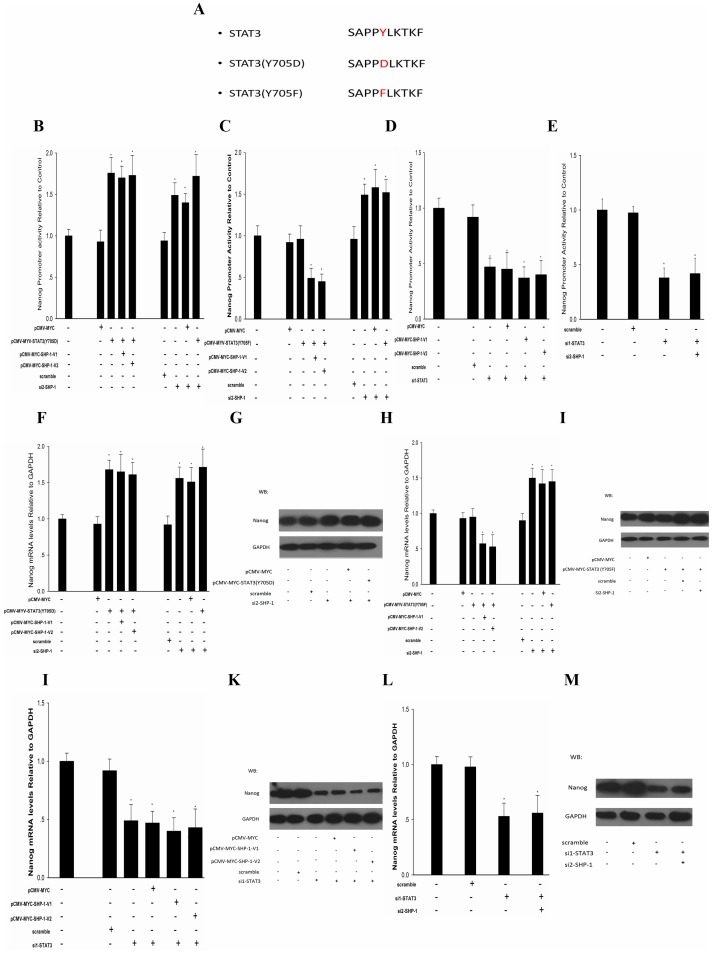
SHP-1 regulates Nanog expression via STAT3 pathway. (A): Schematic representation of STAT3 phospho-mimetic mutant (Y705D) or dominant negative forms of STAT3 (Y705F) constructs. In STAT3 (Y705D) overexpressed F9 cells, overexpress SHP-1 or knockdown SHP-1,DLR assays, qPCR and western blotting were performed to detect the Nanog promoter activity (B) mRNA levels (F) and protein levels (G). In STAT3 (Y705F) overexpressed F9 cells, overexpress SHP-1 or knockdown SHP-1, DLR assays, qPCR and western blotting were performed to detect the Nanog promoter activity (C) mRNA levels (H) and protein levels (I). In knockdown STAT3 F9 cells, overexpress SHP-1, DLR assays, qPCR and western blotting were performed to detect the Nanog promoter activity (D) mRNA levels (J) and protein levels (K). In knockdown STAT3 F9 cells, si SHP-1,DLR assays, qPCR and western blotting were performed to detect the Nanog promoter activity (E) mRNA levels (L) and protein levels (M). All data are presented as the mean +/− SD and are derived from three independent experiments. *P<0.05; **P<0.01; GAPDH was set as loading control. WB: western blot.

With the data above, we found that Nanog expression was up-regulated when SHP-1 was knocked down and down-regulated when SHP-1 was overexpressed. To discover the relationship between SHP-1 and STAT3 in F9 cells, SHP-1 was knocked down or overexpressed in F9 cells that had been transfected with pCMV-MYC-STAT3(Y705D) or pCMV-MYC-STAT3(Y705F) [26], [27], mutated STAT3, phospho-mimetic mutant (Y705D), or dominant negative forms of STAT3 (Y705F),the Schematic representation of STAT3 (Y705D) and STAT3 (Y705F) is shown in Fig. 5. A. In our results, when STAT3 (Y705D) was overexpressed; Nanog promoter activity was up-regulated 1.5–1.6-fold regardless of whether SHP-1 was knocked down or overexpressed (Fig. 5B). However, when STAT3 (Y705F) was overexpressed, knockdown of SHP-1 caused Nanog promoter activity to be up-regulated 1.45-fold, and overexpression of SHP-1 reduced Nanog promoter activity to 50% compared with the control (Fig. 5C). Further more, we found that in STAT3 knocked down F9 cells, Nanog promoter activity was reduced to 50% when overexpressed SHP-1 (Fig. 5D); conversely, Nanog promoter activity was reduced to 42% when SHP-1 was knocked down (Fig. 5E). The RT-PCR and western blotting results also showed that when STAT3 (Y705D) was overexpressed, Nanog was up-regulated at mRNA (1.6–1.7 fold) (Fig. 5F) and protein levels (Fig. 5G) regardless of whether SHP-1 was knocked down or overexpressed; However, when STAT3 (Y705F) was overexpressed, knockdown of SHP-1 caused Nanog to be up-regulated 1.5-fold on mRNA level and overexpression of SHP-1 reduced Nanog mRNA to 50% compared with the control (Fig. 5H), the Nanog protein show the same change (Fig. 5I); in STAT3 knocked down F9 cells, Nanog was reduced to 50% at mRNA level (Fig. 5J) and protein levels (Fig. 5K) when overexpressed SHP-1, Nanog was also reduced to about 50% on mRNA level (Fig. 5L) and protein level (Fig. 5M) when SHP-1 was knocked down. Taken together, all these data revealed that SHP-1 can regulate the expression of Nanog by dephosphorylated STAT3 in F9 cell.

## Discussion

SHP-1 is a 68 kDa protein tyrosine phosphatase comprising two SH2 domains, a tyrosine phosphatase catalytic domain, and a flexible C-terminal domain [24], [28]–[30]. It is an essential regulatory molecule in many signaling pathways involved in differentiation and proliferation [1], [2]. In this study, we found that, in the early period of embryonic development, the SHP-1 expression decreased from1-cell period to 8-cells period, and disappeared in morulae. In contrary, the expression of Nanog cannot be detected until morulae were formed. Moreover, over-expression of SHP-1 in 8-cells embryos led to an inhibition of blastocyst formation and the suppression of Nanog. However, the role of SHP-1 in regulation of early embryonic development is not well defined. Because the limitation of analysis of the molecular mechanisms in embryos, and F9 cell line has been widely used as a model for the analysis of the molecular mechanisms of development and proliferation [31]–[33]. Based on these reasons we use F9 cells as a vitro model to analysis the interaction between SHP-1 and Nanog. Previous reports showed that knockdown of SHP-1 reduced the expression of Nanog from a high level in mouse ES cells [34]. However, the results from our present study indicate that if the SHP-1 is blocked by siRNA, Nanog expression would have a dramatic increase. We hypothesized that the inconsistent may due to the different regulatory networks in different cell lines. With overexpression of STAT3 (Y750F), knockdown of SHP-1 up-regulated Nanog and overexpression of SHP-1 inhibited the expression of Nanog. Nevertheless, with overexpression of STAT3 (Y750D) and STAT3, Nanog expression was up-regulated regardless of the inhibition or overexpression of SHP-1. The explanations to these results are: STAT3 is a member of the STAT protein family, which can be phosphorylated by non-receptor protein tyrosine kinases such as Janus activated kinases. STATs were activated by phosphorylation, and then translocated to nucleus to function as transcription regulators. In this study, STAT3 was inactivated when it was dephosphorylated by SHP-1 or Y750F mutated. Therefore, the overexpressed STAT3 (Y750F) can not be phosphorylated, and Nanog was regulated by SHP-1 directly through endogenous STAT3. Conversely, when large amount of phospho-mimetic STAT3 (Y750D) were overexpressed, Nanog promoter was occupied by activated STAT3 (Y750D), thus Nanog was up-regulated regardless of the inhibition or overexpression of SHP-1.In conclusion, our current results indicate that SHP-1 is a critical regulator of pre-implantation as it is responsible for down-regulation of Nanog expression via STAT3 signaling pathways. Down-regulation of Nanog mRNA expression at implantation is critical to restrict expansion of the epiblast, which coincides with the domain of X chromosome reprogramming. Thus, SHP-1 may function as a key regulator for Nanog which specifically demarcates the nascent epiblast and coincident with the domain of X chromosome reprogramming [8].

## Supporting Information

Table S1 **Sequences of primers for SHP-1 and STAT3.** Restriction sites were underlined.
(DOCX)

Table S2 **Sequences of primers for Real-Time PCR.**
(DOC)

Table S3 **Sequences of primers for Nanog promoters.** Restriction sites were underlined.
(DOC)

Table S4 Sequences of primers for ChIP*-qPCR. *Chromatin immunoprecipitation.
(DOC)
