# Supplementary material for: SHP-1 Arrests Mouse Early Embryo Development through Downregulation of Nanog by Dephosphorylation of STAT3
Source: PLoS One. 2014 Jan 21;9(1):e86330. doi: 10.1371/journal.pone.0086330 (PMC3897670; doi:10.1371/journal.pone.0086330)
Supplement: Table S1 — Sequences of primers for SHP-1 and STAT3. Restriction sites were underlined. (DOCX) [file pone.0086330.s001.docx]

**Table S1 Sequences of primers for SHP-1 and STAT3**

| **Gene Prime name Sequence(5’—3’) Accession number** |
| --- |
| **SHP-1 v1 SHP-1-V1-EcoRI-F AGTGGAATTCCCAGGATGGTGAGGTGGTTTCA NM_013545**  **SHP-1-XhoI-R AGACCTCGAGCCAGATCACTTCCTCTTGAGAG**  **SHP-1 v2 SHP1-V2-EcoRI-F TCCTGAATTCAGATGTTGTCCCGCGGGTGGT NM_001077705**  **SHP-1-XhoI-: AGACCTCGAGCCAGATCACTTCCTCTTGAGAG**  **STAT3 STAT3-F-Bgl II GCTGCGCagatctCTATGGCTCAGTGGAACCAGCTG**  **NM_213659**  **STAT3-R-Not I TAATAgcggccgcCTACATGGGGGAGGTAGCACACT** |

**Restriction sites were underlined**
