# Supplementary material for: SHP-1 Arrests Mouse Early Embryo Development through Downregulation of Nanog by Dephosphorylation of STAT3
Source: PLoS One. 2014 Jan 21;9(1):e86330. doi: 10.1371/journal.pone.0086330 (PMC3897670; doi:10.1371/journal.pone.0086330)
Supplement: Table S2 — Sequences of primers for Real-Time PCR. (DOC) [file pone.0086330.s002.doc]

**Table S2** Sequences of primers for Real-Time PCR

| **Gene Primer name Sequence(5’—3’) Product length**  **(Accession number)** |
| --- |
| **GAPDH F GTGTTCCTACCCCCAATGTGT 248 bp**  **(NM_008084) R ATTGTCATACCAGGAAATGAGCTT**  **Nanog F CACCCACCCATGCTAGTCTT 150bp**  **(****NM_028016) R ACCCTCAAACTCCTGGTCCT** |
