# Supplementary material for: SHP-1 Arrests Mouse Early Embryo Development through Downregulation of Nanog by Dephosphorylation of STAT3
Source: PLoS One. 2014 Jan 21;9(1):e86330. doi: 10.1371/journal.pone.0086330 (PMC3897670; doi:10.1371/journal.pone.0086330)
Supplement: Table S3 — Sequences of primers for Nanog promoters. Restriction sites were underlined. (DOC) [file pone.0086330.s003.doc]

**Table S3** Sequences of primers for Nanog promoters

| **Primer name Primer sequence(5′–3′) Product length** |
| --- |
| **NP-1.0k-F-Xho I CTT CTCGAGCTTAGACGGCTGAGGCACTT 1064 bp**  **NP-R-Bgl II CCG AGATCTGCATTGATGAGGCGTTCC**  **STAT3 site-WT-F GCC GGTACCAGAGGACTCGCATGCATTTTG 234bp**  **STAT3 site-WT-R CTT CTCGAGCCAGCTCTGCCCTTACAAAAA** |

**Restriction sites were underlined**
