# Supplementary material for: SHP-1 Arrests Mouse Early Embryo Development through Downregulation of Nanog by Dephosphorylation of STAT3
Source: PLoS One. 2014 Jan 21;9(1):e86330. doi: 10.1371/journal.pone.0086330 (PMC3897670; doi:10.1371/journal.pone.0086330)
Supplement: Table S4 — Sequences of primers for ChIP*-qPCR. *Chromatin immunoprecipitation. (DOC) [file pone.0086330.s004.doc]

**Table S4 Sequences of primers for ChIP＊**-qPCR

| **Prime name Sequence(5’—3’) Product length** |
| --- |
| **STAT3-ChIP-F TGGGGTAAACTTAAGGCTATGGTGG 219 bp**  **STAT3-ChIP-R CCAAGGGCGACGTAATTTTGGTA**  **Control-F TGTGACTAACCTCATTCCTC 243 bp**  **Control-R CTACGCCATAACTACCTCTG** |

**＊Chromatin immunoprecipitation**
